# The Genetic Mosaic of Depression: Linking Polymorphisms to Neuroplasticity and Stress Regulation

**DOI:** 10.3390/ph19020336

**Published:** 2026-02-20

**Authors:** Aneta Bednářová, Emma Szilassyová, Dominika Jarčušková, Daniel Múdry, Terézia Kisková-Šimková

**Affiliations:** 1 2nd Department of Psychiatry, Faculty of Medicine, Pavol Jozef Šafárik University and Louis Pasteur University Hospital, Rastislavova 43, 04190 Košice, Slovakia; emma.szilassyova@upjs.sk; 2 1st Department of Psychiatry, Faculty of Medicine, Pavol Jozef Šafárik University and Louis Pasteur University Hospital, Trieda SNP 1, 04011 Košice, Slovakia; 3 B.Elite, Trieda SNP 339/88, 04011 Košice, Slovakia; 4 Department of Pathology, Faculty of Medicine, Pavol Jozef Šafárik University, Rastislavova 43, 04190 Košice, Slovakia

**Keywords:** major depressive disorder (MDD), depression, genetic polymorphisms, genetics, heritability, psychiatric genetics

## Abstract

The origins of major depressive disorder (MDD) are complex, involving both environmental influences and a substantial genetic contribution. Genetic polymorphisms have been implicated in modulating susceptibility, disease course, and treatment response, yet findings are often modest, population-dependent, and sometimes inconsistent. This narrative review synthesizes current evidence on genetic variants associated with MDD, highlighting well-replicated results while distinguishing exploratory or emerging findings. Key systems reviewed include serotonergic (*SLC6A4*), neurotrophic (*BDNF* rs6265 and rs962369), dopaminergic and stress-response pathways (*COMT*, *FKBP5*, *CRHR1*), as well as additional emerging genes such as *MAOA*, *TPH2*, and *FTO*. We evaluate these variants in the context of their biological relevance, including neuroplasticity, neurotransmission, and hypothalamic–pituitary–adrenal (HPA) axis regulation, and discuss how polygenic and epigenetic interactions may shape clinical heterogeneity. This framework not only integrates current genetic knowledge but also outlines potential translational applications, offering perspectives for personalized approaches to diagnosis, prognosis, and treatment in MDD.

## 1. Introduction

Major depressive disorder (MDD), commonly referred to as depression, is a chronic and recurrent psychiatric illness and a leading cause of disability worldwide [1]. It is characterized by a heterogeneous constellation of affective, cognitive, behavioral, and somatic symptoms, including persistent sadness, anhedonia, psychomotor changes, sleep and appetite disturbances, impaired concentration, fatigue, and suicidality [1,2]. Depressive symptoms are not exclusive to MDD but may also occur in bipolar disorder (BD), where depressive episodes can be recurrent and clinically indistinguishable from those in MDD. In some patients, BD type II may be misdiagnosed as MDD due to infrequent or subtle hypomanic episodes, highlighting challenges in diagnosis and implications for both research and clinical management [2]. Globally, approximately 3.8% of the population suffer from depression, including about 5% of adults and 5.7% of those over 60 years old [3,4].

Depression results from complex interactions among genetic, environmental, and neurobiological factors. Classical hypotheses include dysregulation of central monoamine neurotransmitters—serotonin, norepinephrine, and dopamine—which influence mood, cognition, and motivation [5,6]. Chronic low-grade inflammation, immune activation, and HPA axis dysregulation further modulate disease vulnerability, linking stress exposure to glucocorticoid imbalance, altered neuroplasticity, and impaired feedback regulation [7,8,9,10]. Genetic predisposition interacts with these biological systems and environmental stressors, shaping individual susceptibility across the lifespan [11,12,13]. Specific genetic variants influence neurotransmission, neuroplasticity, stress responsivity, and immune signaling, while adverse life events, early trauma, and chronic stress may act as precipitating or perpetuating factors [14].

The progression of ever more detailed diagnostic tools (DSM-5R; ICD-11) [15,16] is contributing to better distinguish depression and to identify patient subtypes with distinct symptom clusters, modifying epidemiological indices and making fertile backgrounds for genetic and epigenetic studies. Family and twin studies indicate a heritable component of MDD, with estimates around 37%, and first-degree relatives of affected individuals having a two- to three-fold increased risk [12]. Molecular genetics, including Genome-Wide Association Study (GWAS), mendelian randomization studies, and candidate gene studies, have identified numerous Single Nucleotide Polymorphisms (SNPs) associated with MDD [13]. However, effect sizes are generally modest, findings are often population-dependent, and replication across studies is inconsistent [2]. These observations underscore the polygenic and multifactorial nature of depression, highlighting the need to interpret genetic associations within broader biological and environmental contexts.

This narrative review focuses on selected functional polymorphisms—such as the Serotonin-transporter-linked polymorphic region (5-HTTLPR), Catechol-O-methyltransferase (*COMT*) Val158Met, and Brain-derived neurotrophic factor (*BDNF*) Val66Met—chosen for their biological relevance, reproducibility, and central roles in monoaminergic neurotransmission, neuroplasticity, and stress regulation [17,18,19]. Additional variants are discussed to illustrate the heterogeneous genetic architecture of MDD. By integrating genetic evidence with mechanistic insights and considering gene-environment and epigenetic interactions, this review provides a framework for understanding clinical heterogeneity and exploring personalized strategies for prevention, diagnosis, and treatment.

## 2. Materials and Methods

This narrative review is based on a structured literature search of genetic studies on MDD published between 1 January 2000 and 30 November 2025 in the PubMed database. Two investigators independently conducted the search using combinations of the following keywords: “major depressive disorder” OR “depression” AND “gene candidate,” and “major depressive disorder” OR “depression” AND “genetic polymorphism.”

Eligible studies met the following criteria: (i) participants had a primary diagnosis of MDD; (ii) the study investigated associations between genetic polymorphisms and MDD; and (iii) the article was published in English. Exclusion criteria were: (i) non-human studies; (ii) non-genetic studies; (iii) studies including participants without a primary MDD diagnosis or with major psychiatric comorbidities; and (iv) articles with insufficient data (e.g., abstract only).

While this review is narrative in nature, the structured search strategy allowed for systematic identification of relevant literature. Emphasis was placed on selecting studies that provide mechanistic insights or replicated associations, enabling a synthesis of patterns, consistencies, and key contradictions across the field rather than a simple cataloging of findings.

### 2.1. Variants in the Serotonergic System

Serotonin (5-hydroxytryptamine, 5-HT) is a key neurotransmitter involved in regulating mood, emotional behavior, and cognitive functions. Dysregulation of the serotonergic system is closely linked to the pathophysiology of MDD and represents a primary target for pharmacotherapy, particularly selective serotonin reuptake inhibitors (SSRIs) [5].

One of the most extensively studied genetic variants in this system is the 5-HTTLPR polymorphism in the Solute Carrier Family 6 Member 4 (*SLC6A4*) gene, which encodes the serotonin transporter (SERT). Meta-analyses including over 1400 subjects have shown associations between 5-HTTLPR variants and antidepressant response, although results are heterogeneous [20,21,22]. Functional modulation by additional SNPs such as rs25531 has led to a triallelic model: Long allele with adenine at rs25531 (La), Long allele with guanine at rs25531 (Lg), and Short allele (S), which affects transporter expression and potentially treatment outcomes [23].

Other serotonergic genes, including those encoding various 5-HT receptors and enzymes involved in serotonin synthesis, also contribute to MDD susceptibility and influence individual responses to treatment. Variants in these genes can affect receptor function, neurotransmission efficiency, and downstream signaling pathways, thereby impacting mood regulation and treatment efficacy. Table 1 summarizes key serotonergic polymorphisms and their functional relevance.

In addition to serotonergic genes, Table 1 also includes polymorphisms in the kynurenine pathway, which competes with tryptophan hydroxylase-mediated serotonin biosynthesis and has been implicated in depression. This highlights that multiple metabolic and neurotransmitter pathways converge on serotonin availability and function, contributing to the polygenic architecture of MDD [24].

The serotonin system mediates its effects through multiple receptor subtypes, organized into seven major classes, each with further subdivision [25]. Polymorphisms in these receptor genes can influence receptor expression, function, and downstream signaling, thereby modulating mood regulation, stress responsiveness, and antidepressant efficacy. The 5-HT1A receptor, encoded by an intronless gene, is a key autoreceptor that regulates serotonin release and neuronal activation [26]. This metabotropic 5-HT receptor subtype can also be located at the postsynaptic level in specific brain areas [27]. Although predominantly linked to Inhibitory G protein (Gi/o) protein coupling, adenylate cyclase inhibition and ion channel regulation, this 5-HT receptor has been found to display a complex signal transduction pattern also depending on the brain region [27,28]. Both pre- and post-synaptic 5-HT1A receptors have been involved in mood and anxiety regulation. The rs6295 polymorphism in its promoter region affects receptor expression and may influence susceptibility to depression [29].

The 5-HT2A receptor acts primarily postsynaptically and is highly expressed in cortical and limbic regions, including the prefrontal cortex and hippocampus. Through coupling to Gq/11 proteins, 5-HT2A activation modulates glutamatergic and dopaminergic neurotransmission, influencing emotional processing, cognition, and sleep–wake cycles. Dysregulation of 5-HT2A signaling has been associated with affective symptoms, cognitive alterations, and variability in antidepressant treatment response [30,31]. Large pharmacogenetic studies suggest that HTR2A variants may modestly influence antidepressant response, while the effects are context- and population-dependent [32,33,34,35]. Certain variants, such as rs7997012 and rs6314, may also impact tolerability [36,37].

The 5-HT1B receptor functions as both an autoreceptor and heteroreceptor, modulating serotonin and other neurotransmitter releases. Altered receptor function may contribute to dysregulated serotonergic tone in depression [38]. Serotonin synthesis begins with hydroxylation of tryptophan-by-tryptophan hydroxylase enzymes encoded by Tryptophan hydroxylase *(TPH)1* and *TPH2. TPH1* is mainly expressed peripherally but also in the pineal gland. Functional polymorphisms in TPH genes can influence antidepressant response and stress sensitivity [38]. For example, the *TPH1* 218C/C genotype was associated with improved response in seven studies including 754 subjects [39,40,41,42,43,44,45], whereas *TPH2* SNPs (rs1843809, rs1386492, rs1487276) showed marginal associations with fluoxetine response [46].

Beyond genetic variants, epigenetic regulation, particularly DNA methylation of the *SLC6A4* promoter, plays a crucial role. Increased methylation can reduce gene expression, alter serotonergic neurotransmission and increase depression risk [5]. This exemplifies the gene–environment interplay, where genetic polymorphisms, epigenetic modifications, and environmental exposures collectively modulate individual vulnerability to MDD.

Integrating findings across serotonergic genes and related pathways demonstrates that depression risk and treatment response are shaped by polygenic, epigenetically modulated mechanisms. The interplay of multiple genes, environmental influences, and regulatory mechanisms supports a framework in which clinical heterogeneity in MDD emerges from complex molecular networks rather than single gene effects.

An overview of key serotonergic and related polymorphisms, their functional effects, and clinical implications is provided in Table 2.

### 2.2. Variants in Neurotrophic Signaling

Brain-derived neurotrophic factor (*BDNF*) is the most important neurotrophin, supporting neuronal survival, synaptic plasticity, and neurogenesis. Reduced *BDNF* expression has been consistently reported in patients with MDD. It is associated with synaptic dysfunction and atrophy of brain regions such as the hippocampus and prefrontal cortex [47]. Antidepressants increase *BDNF* expression, which supports the so-called neurotrophic hypothesis of depression [48]. Recently, *BDNF* gene polymorphisms have been reported to play important roles in depression and cognitive impairment [7].

The most studied genetic variant is the SNP rs6265, which encodes a Valine-to-Methionine substitution at codon 66 (Val66Met) [49]. Substitution of valine by methionine at codon 66 leads to reduced activity and secretion of *BDNF* [47]. This variant is associated with increased susceptibility to depression, changes in the hippocampus, and poorer treatment response. However, findings across studies remain heterogeneous, suggesting that Val66Met acts as a context-dependent risk modifier rather than a deterministic factor, with effects influenced by population characteristics, environmental stressors, and interactions with other genetic variants [8,50]. Consistent with this view, Colle et al. reported that carriers of the Met allele exhibited significantly lower plasma BDNF levels compared with Val/Val homozygotes, but they also observed that BDNF levels were more associated with clinical characteristics and course of major depressive disorder in Met carriers following an unusual pattern, reflecting that peripheral BDNF may reveal compensatory attempts to restrain the lower levels of this neurotrophin [51].

Several studies have explored the role of Val66Met in antidepressant treatment outcomes. Colle et al. reported differential treatment trajectories depending on antidepressant class, with Val/Val carriers showing a more favorable early response to selective serotonin reuptake inhibitors, but lower long-term remission rates with serotonin–norepinephrine reuptake inhibitors or tricyclic antidepressants [52]. These findings suggest that Val66Met does not confer a uniform treatment advantage or disadvantage but rather modulates treatment response patterns. Consistent with this interpretation, a meta-analysis by Kato and Serretti observed improved antidepressant response among carriers of the Met allele [39]. Importantly, interpretation of these findings is limited by the relatively short duration of most available trials (typically 4–8 weeks), whereas BDNF-mediated neurogenic and synaptic effects may require longer treatment periods to become clinically evident. [53].

Beyond rs6265, increasing attention has been directed toward the *BDNF* rs962369 polymorphism. Emerging evidence indicates that the rs962369 C allele is associated with an elevated risk of both single-episode and recurrent depression, with a dose-dependent relationship reported in recurrent forms of the disorder [54]. These findings further support the notion that multiple *BDNF* variants contribute to depression susceptibility and clinical heterogeneity.

Although rs6265 and rs962369 are among the best characterized *BDNF* polymorphisms, numerous additional variants are likely to influence neurotrophic signaling by modulating *BDNF* expression, receptor interactions, or downstream neuroplastic processes. Collectively, these genetic differences may shape individual vulnerability to depression as well as variability in clinical course and treatment outcomes. Additional BDNF variants investigated in this context are summarized in Table 3.

BDNF signaling is primarily mediated by the tropomyosin receptor kinase B (TrkB), encoded by the Neurotrophic receptor tyrosine kinase 2 (*NTRK2*) gene. Polymorphisms in *NTRK2* have been associated with increased risk of depression and self-harm, underscoring the importance of intact *BDNF*–TrkB signaling for neuroplastic resilience [55].

Disruptions in this pathway may impair synaptic maintenance and promote apoptotic processes. Moreover, psychosocial stress has been shown to reduce *BDNF* levels and induce epigenetic modifications that may further compromise neurotrophic signaling [9].

Interactions between *BDNF* polymorphisms and variants in other systems, such as the serotonin transporter (e.g., 5-HTTLPR), highlight the complex gene–environment interplay underlying depression risk, particularly following exposure to stress.

Finally, genetic variation within the broader neurotrophic network also appears to influence antidepressant efficacy. For example, polymorphisms in *BDNF* and vascular endothelial growth factor A (*VEGFA*) genes have been associated with differential therapeutic responses, further supporting the role of neuroplastic mechanisms in treatment outcomes [34].

### 2.3. Variants in Dopaminergic and Stress-Response Pathways

The dopaminergic system and stress-response pathways play central roles in mood regulation, motivation, and resilience to psychosocial stress [56]. Their functional interaction represents a core component of the neurobiological framework underlying stress-related psychiatric disorders, including MDD. Accumulating evidence indicates that genetic variation within catecholaminergic and HPA axis pathways contributes to individual differences in stress susceptibility, clinical presentation, and treatment response [57,58,59]. In particular, polymorphisms in genes such as *COMT*, FK506 binding protein 5 (*FKBP5*), and Corticotropin-releasing hormone receptor 1 (*CRHR1*) have been consistently implicated in depression pathophysiology and stress responsivity [18].

The catechol-O-methyltransferase (*COMT*) gene encodes an enzyme responsible for the degradation of catecholamines, including dopamine, norepinephrine, and epinephrine, and plays a critical role in regulating dopamine availability in the prefrontal cortex [60]. Genetic variation in *COMT* influences synaptic dopamine levels, cognitive performance, emotional regulation, and vulnerability to psychiatric disorders [61]. The most extensively studied polymorphism, Val158Met, results in a valine-to-methionine substitution that alters enzymatic activity. The Val allele is associated with higher *COMT* activity and faster dopamine catabolism, whereas the Met allele confers reduced enzymatic activity and higher cortical dopamine levels [62,63]. These functional differences have been linked to variability in executive function, working memory and attentional processes, which are frequently impaired in depression [64]. Individuals with the Val/Val genotype tend to have lower dopamine levels and have been associated with a higher risk of depression and poorer response to antidepressant treatment [65]. Several studies suggest that the Val/Val genotype may be associated with increased depression risk and less favorable antidepressant response, particularly during early treatment phases [65,66]. In contrast, the Met/Met genotype is associated with better cognitive outcomes and may show differential responses to interventions such as electroconvulsive therapy (ECT), transcranial magnetic stimulation (TMS), and dopaminergic agents [67]. However, findings remain inconsistent, and some studies report no significant effect of *COMT* genotype on antidepressant efficacy [68]. These discrepancies suggest that *COMT* Val158Met acts as a modulatory factor whose clinical relevance depends on treatment type, disease characteristics, and interactions with other genetic and environmental influences [69,70,71,72]. Beyond dopamine metabolism, genetic variation in dopamine receptors and transporters further contributes to depression vulnerability. Dopamine receptor subtypes (*DRD2*, *DRD3*, *DRD4*) and the dopamine transporter (DAT), encoded by Solute carrier family 6 member 3 (*SLC6A3*), are critical regulators of reward processing, motivation, and mood [73]. A recent comprehensive review highlights the role of dopamine receptor heterodimers, particularly D1–D2 complexes, in affective regulation [32]. Although heterodimer formation is not genetically encoded per se, polymorphisms in *DRD1* and *DRD2* may influence receptor expression and signaling efficiency. Experimental studies suggest that increased D1–D2 heterodimer activity is associated with depressive-like phenotypes, whereas disruption of these complexes reduces depressive behaviors, identifying them as potential therapeutic targets [32].

Alterations in *DRD3* signaling have also been consistently linked to depression and anxiety. Reduced *DRD3* expression induces chronic depressive-like symptoms in animal models independent of motor dysfunction, and antidepressant treatment appears to normalize *DRD3* levels, supporting its relevance for emotional regulation and treatment response [74]. In addition, variants in genes involved in dopamine biosynthesis and transport contribute to interindividual differences in dopaminergic tone and depression risk [75]. Notably, *DRD3* polymorphisms have been associated with differential response to electroconvulsive therapy in treatment-resistant depression, further supporting its role as a candidate gene in pharmacogenetic research [76].

The dopamine transporter (DAT), encoded by the *SLC6A3* gene, regulates synaptic dopamine availability by mediating dopamine reuptake into presynaptic neurons [77]. The most studied *SLC6A3* variant is a variable number tandem repeat (VNTR) polymorphism in the 3′ untranslated region, which affects DAT expression and dopamine signaling in brain regions implicated in mood regulation, including the prefrontal cortex and striatum. A 2023 review found that the SS (9R/9R) genotype and S (9R) allele are linked to increased risk of developing MDD and may influence a poorer response to antidepressant therapy, SSRIs [78]. However, results remain heterogeneous across populations, underscoring the need for further large-scale and ethnically diverse studies [79,80]. Collectively, available data support *SLC6A3* as a modulatory gene influencing depressive symptom severity, cognitive deficits, and antidepressant efficacy, with potential relevance for personalized treatment strategies [78,79,81].

Dysregulation of the HPA axis represents a hallmark of stress-related psychiatric disorders. *FKBP5* encodes FK506 binding protein 51 (FKBP51), a co-chaperone that regulates glucocorticoid receptor (GR) sensitivity and termination of the stress response. By impairing GR nuclear translocation and weakening cortisol negative feedback, increased *FKBP51* activity leads to prolonged HPA axis activation, a pattern observed in some forms of Post-traumatic stress disorder (PTSD) and MDD [10].

Genetic variants in *FKBP5*, including rs1360780 and rs3800373, have been repeatedly associated with altered cortisol dynamics and increased stress sensitivity. Carriers of *FKBP5* risk alleles often exhibit impaired cortisol recovery after stress exposure, heightened anxiety, and dysregulated HPA axis feedback, with some effects showing sex-specific patterns [82,83]. At the cellular level, *FKBP5* expression in the paraventricular nucleus of the hypothalamus critically modulates GR signaling in corticotropin-releasing hormone neurons, thereby shaping acute and chronic stress responses [84]. Epigenetic modifications of *FKBP5*, particularly stress-induced DNA demethylation following early-life adversity, may produce long-lasting alterations in HPA axis regulation and increase vulnerability to depression and related disorders [85,86]. These findings position *FKBP5* as a key molecular integrator of genetic and environmental stress signals. Dysregulation of this system contributes to the pathophysiology of depression, PTSD, and other stress-related disorders [87]. These findings position *FKBP5* as a promising biomarker and therapeutic target for improving stress resilience and developing personalized treatments for psychiatric illnesses [88].

The Corticotropin-releasing hormone receptor 1 (*CRHR1*) gene encodes the *CRHR1*, a key receptor initiating HPA axis activation in response to stress [89]. Variants in *CRHR1* influence individual sensitivity to stress and modulate the risk of depression and PTSD, especially when interacting with early-life adversity such as childhood maltreatment [90]. Certain *CRHR1* haplotypes appear to have a protective effect against depression in maltreated individuals, possibly by affecting emotional memory consolidation. Additionally, *CRHR1* variants are linked to altered cortisol responses to stress and influence antidepressant treatment response, including to drugs like tianeptine [91,92].

Stress susceptibility and resilience are shaped by the interplay among genetic variants, epigenetic modifications (e.g., DNA methylation, miRNA changes), and hormonal regulation [93]. These multilayered mechanisms modulate neuroendocrine, synaptic, and immune pathways implicated in MDD [14]. Emerging evidence shows that stress-induced epigenetic changes, such as MicroRNA (miRNA) alterations in sperm, can be inherited, influencing offspring vulnerability to depression. Sex-specific molecular pathways, including estrogen’s neuroprotective role, further modulate these effects [93]. The polygenic nature of depression involves multiple small-effect variants interacting with environmental stressors. Genome-wide studies emphasize the importance of gene-gene and gene-environment interactions in shaping stress response and depression phenotypes [14].

### 2.4. Emerging Genetic Variants and New Target Genes

New variants of genes already investigated for association with depressive and psychiatric symptoms, such as monoamine oxidase A (*MAO-A*) and Tryptophan hydroxylase-2 (*TPH-2*), are being now evaluated for their particular importance in this area, along with other gene variants that are relatively new in the field of etiopathogenetic studies of depression, such as Fat mass and obesity-associated (*FTO*) and the gene encoding the Piccolo Presynaptic Cytomatrix Protein (*PCLO*) [94].

Monoamine Oxidase A (*MAOA*) is a mitochondrial enzyme that degrades key neurotransmitters, including serotonin, norepinephrine, and dopamine, all of which are critical for mood regulation [95]. The *MAOA* gene is located on the X chromosome (Xp11.23) [38]. A key functional polymorphism in the *MAOA* gene promoter region is the upstream variable number tandem repeat (uVNTR), consisting of 30-base pair repeats with alleles ranging from 2 to 6 [94]. The most studied alleles are the 3R allele, which is associated with low transcriptional activity and lower *MAOA* enzyme expression and the 3.5R and 4R alleles, which are associated with higher transcriptional activity and higher enzyme expression. This polymorphism influences *MAOA* enzyme levels and thus monoamine metabolism, potentially affecting susceptibility to MDD [96].

A 2022 systematic review analyzed seven eligible studies investigating the association between *MAOA* uVNTR genotypes and MDD across diverse populations, predominantly Asian. Key findings include a higher frequency of the 3R/3R genotype in females and the 3R genotype in males among healthy control groups. Conversely, the 4R/4R genotype in females and the 4R genotype in males were more common in patients with MDD. These patterns suggest that the high-activity 4R allele may increase the risk of MDD, whereas the low-activity 3R allele may confer a protective effect [97].

However, results across studies were not entirely consistent, and no definitive consensus has been reached regarding the role of *MAOA* uVNTR in MDD pathogenesis. Alterations in *MAOA* activity may contribute to the neurochemical imbalance observed in depression. Understanding these genetic influences could improve the identification of individuals at higher risk and inform personalized treatment strategies.

While the *MAOA* uVNTR polymorphism is a promising candidate influencing MDD susceptibility, current evidence remains inconclusive due to limited and heterogeneous data. Future research should focus on larger, ethnically diverse cohorts with standardized phenotyping and consider gene-environment interactions. Integrating *MAOA* genotyping into clinical practice may eventually help tailor antidepressant therapy and improve outcomes for patients with MDD [97].

Tryptophan Hydroxylase 2 (*TPH2*) is a brain-specific isoenzyme responsible for the rate-limiting step in serotonin synthesis, converting tryptophan to 5-hydroxytryptophan [98]. It has been extensively linked to MDD [99]. Serotonin (5-HT) plays a pivotal role in mood regulation [100]. Abnormalities in its synthesis and signaling are central to MDD pathophysiology [101]. Genetic studies have shown that polymorphisms in the *TPH2* gene are associated with susceptibility to MDD and influence antidepressant treatment response [102,103]. For example, a study involving a Chinese population found that the rs17110747-G homozygous variant was more frequent in MDD patients than controls [104]. Moreover, carriers of the rs2171363 heterozygote variant exhibited a better therapeutic response to SSRIs such as fluoxetine or citalopram after eight weeks of treatment [105]. This suggests that *TPH2* genetic variation can modulate both disease risk and clinical outcomes in MDD patients [105]. Beyond genetics, stress can epigenetically regulate *TPH2* expression. Experimental rat models of depression showed stress-induced repression of *TPH2* expression in the brain, accompanied by increased methylation of the *TPH2* gene promoter region [35,106]. Such epigenetic modifications reduce serotonin biosynthesis, contributing to depressive phenotypes. Notably, treatment with the SSRI paroxetine reversed these effects by restoring *TPH2* expression and reducing promoter methylation, highlighting a link between stress, *TPH2* regulation, serotonin synthesis, and antidepressant efficacy [106]. *TPH2* also plays a key role in the integration of the serotonergic system with the HPA axis stress response [107]. Upon stress-induced cortisol release, cortisol can induce *TPH2* expression and serotonin synthesis, which in turn feedback to modulate cortisol secretion through complex neuroendocrine pathways. Variations in *TPH2* regulatory elements may thus differentially influence stress reactivity and vulnerability to stress-related disorders such as depression [106]. Further studies suggest some *TPH2* variants might provide protective effects. Especially in certain subgroups such as females, these studies indicate that the genetic association with MDD is nuanced and likely influenced by sex and other factors [106,108]. In summary, *TPH2* is critically implicated in MDD through its impact on brain serotonin synthesis [109]. Genetic polymorphisms and epigenetic regulation of *TPH2* affect individual susceptibility to depression and modulate response to antidepressants, particularly SSRIs. *TPH2*’s role in the stress-serotonin-HPA axis interplay underscores its importance as a therapeutic and research target in MDD [105,110].

The *FTO* gene (Fat Mass and Obesity-Associated Gene) encodes a protein involved in RNA demethylation, influencing various physiological and neural functions [111]. While *FTO* is most recognized for its association with obesity and higher body mass index (BMI), emerging evidence implicates *FTO* in the pathophysiology of MDD, especially in the context of obesity-depression comorbidity [112]. *FTO* is highly expressed in the brain, particularly in regions such as the hypothalamus and hippocampus, which are critical for regulating mood and metabolic processes [113]. Through its enzymatic activity, *FTO* modulates RNA methylation status (especially N6-methyladenosine, or m6A), influencing gene expression relevant to neuronal signaling and synaptic plasticity [113]. In depression, hippocampal *FTO* expression is notably reduced. Animal models show that knockdown or knockout of *FTO* in the hippocampus induces depression-like behaviors, whereas overexpression has demonstrated antidepressant effects [112].

Multiple studies and meta-analyses confirm that depression moderates the impact of *FTO* risk alleles on BMI, with risk allele carriers who have MDD experiencing greater BMI increases compared to controls without depression. This indicates that depression amplifies *FTO*’s effect on obesity, providing a biological basis for the high comorbidity between these conditions [114]. Contrary to some expectations, other studies indicate that certain *FTO* gene variants may confer a modest reduction in depression risk, up to 8% in large international cohorts. This “happy gene” hypothesis suggests a nuanced interaction between *FTO*, metabolic regulation, and mood, hinting at a potential protective mechanism independent of BMI [115]. *FTO* regulates m6A demethylation on RNA transcripts, including those for adrenergic receptors, which are implicated in neural plasticity and emotional processing [113]. Loss of *FTO* in the hippocampus increases m6A modification and reduces expression of critical genes, contributing to depressive behaviors [112]. Restoration of *FTO* activity or downstream targets exhibits antidepressant-like effects in animal models [116]. Some research suggests that the relation between *FTO*, BMI, and MDD may be specific to certain depression subtypes, especially those associated with atypical features and a greater risk of weight gain. More precise clinical phenotyping is needed to map these associations [117,118]. Studies directly linking *FTO* variants to depression in the absence of obesity are relatively scarce, and findings are sometimes conflicting, indicating the necessity for more targeted research in comorbid samples and across depression subtypes [119]. Future studies should consider depression subtypes, metabolic status, and longitudinal trajectories to clarify the role of *FTO* in mood disorders [120].

The *PCLO* gene (Piccolo Presynaptic Cytomatrix Protein) encodes a large presynaptic scaffolding protein that plays a critical role in synaptic vesicle trafficking, active zone organization [117,118], and efficient neurotransmitter release, particularly in monoaminergic synapses relevant to mood regulation [121]. Large-scale genome-wide association studies have consistently identified *PCLO* variants as genetic risk loci for MDD, supporting the involvement of presynaptic dysfunction in depression pathophysiology [11,122]. Alterations in *PCLO* function may impair synaptic plasticity and neurotransmission efficiency, thereby affecting emotional processing and stress responsiveness. Network-based genomic analyses further suggest that *PCLO* is part of a broader synaptic gene cluster implicated in depression and related affective disorders, highlighting the importance of synaptic organization and signaling in MDD vulnerability [121,122].

Across the genetic pathways discussed in this review, including serotonergic, dopaminergic, neurotrophic, metabolic, and stress-response systems, a common mechanistic theme emerges in which genetic variants exert their effects through epigenetic regulation and context-dependent changes in gene expression. Key genes such as *SLC6A4*, *BDNF*, *COMT*, *FKBP5*, *CRHR1*, *TPH2*, *FTO*, and *PCLO* act as molecular integrators of environmental exposures—particularly stress, metabolic imbalance, and inflammation, thereby shaping neurotransmission, neuroplasticity, and stress responsivity. This genetic–epigenetic–transcriptional continuum provides a unifying framework for understanding the marked clinical heterogeneity of major depressive disorder and underscores the importance of pathway-based and personalized approaches to diagnosis, prognosis, and treatment.

## 3. Discussion

Recent research shows that MDD arises from the interaction of multiple genetic variants and biological processes [11]. This review highlights the complex interplay between genetic polymorphisms, neurobiological systems, and environmental influences in shaping individual vulnerability to depression. The available genetic evidence ranges from robust findings replicated across multiple studies to more tentative associations that require further validation. Distinguishing between these levels of evidence is essential for interpreting their biological relevance and potential clinical applicability. Among the most extensively studied contributors to MDD are genes involved in serotonergic neurotransmission. Variants within this system remain central to understanding the neurochemical basis of depression. The 5-HTTLPR polymorphism in *SLC6A4* is one of the most frequently investigated genetic variants and has been repeatedly associated with increased susceptibility to depression and variability in antidepressant response. Nevertheless, findings remain partly inconsistent, largely due to population heterogeneity and gene–environment interactions [123]. Despite this variability, alterations in *SLC6A4*-related signaling consistently emerge across studies, suggesting that the serotonergic pathway is better conceptualized as a reliable biological system rather than as a source of single, definitive biomarkers. Similarly, polymorphisms in *HTR2A* and *HTR1A* show potential value in predicting antidepressant efficacy and tolerability, underscoring the functional importance of receptor density and signaling dynamics [124]. Variants in *TPH2*, which influence serotonin, further integrate serotonergic function with HPA axis activation, thereby linking neurotransmission with stress responsiveness [98]. Overall, serotonergic genes are supported by relatively strong evidence; however, their individual effects are small and highly context dependent. Consequently, they are most meaningfully interpreted as part of a broader genetic network rather than as isolated variants.

Beyond monoaminergic signaling, neurotrophic factors play a critical role in the pathophysiology of MDD. *BDNF* and its receptor TrkB, encoded by *NTRK2*, are key regulators of synaptic plasticity, neuronal survival, and resilience to stress [125]. The Val66Met polymorphism in *BDNF* has been associated with hippocampal function, stress sensitivity, and treatment response [50]. In addition, the more recently described rs962369 polymorphism may represent a stronger predictor of recurrent depressive episodes, suggesting that the combined assessment of multiple *BDNF* variants could enhance personalized treatment strategies [126]. Compared with monoaminergic genes, neurotrophic pathways may provide greater biological specificity with respect to illness course and recurrence, positioning them as potential prognostic rather than diagnostic biomarkers. However, current findings remain inconsistent and context dependent, and these markers have not yet been validated for routine clinical use.

Genetic variation within dopaminergic pathways further contributes to the neurobiological heterogeneity of MDD. Polymorphisms in *COMT*, *DRD2*, *DRD3*, and *SLC6A3* have been linked to alterations in dopamine metabolism, cognitive performance, reward processing, and antidepressant responsiveness [73]. The *COMT* Val158Met variant and the VNTR polymorphism in *SLC6A3* are among the most studied examples and have shown associations with executive function and treatment outcomes [79]. Emerging evidence suggests that dysregulation of D1–D2 receptor heterodimer function and altered D3 receptor expression may represent novel therapeutic targets, particularly in treatment-resistant depression [73]. Nonetheless, these findings remain heterogenous, and their clinical relevance should be interpreted cautiously until further replicated. A defining characteristic of MDD is its pronounced clinical heterogeneity, encompassing variability in symptom presentation, illness severity, recurrence, and response to treatment. This heterogeneity likely reflects the cumulative effect of polygenic vulnerability, whereby numerous common genetic variants of small effect interact across multiple neurobiological systems [11]. Depression is a highly polygenic and heterogeneous disorder, with risk distributed across thousands of common genetic variants of small effects. Polygenic risk scores reveal substantial interindividual variability, reflecting distinct biological pathways involving neurodevelopment, stress reactivity, immune signaling, circadian regulation, and metabolic function. This polygenic architecture contributes to clinical heterogeneity in symptom profiles, age of onset, comorbidity patterns, and treatment response, underscoring the need for genetically informed subtyping and precision psychiatry approaches. Importantly, the functional consequences of these variants are strongly shaped by epigenetic mechanisms that regulate gene expression in response to environmental exposures, including early-life adversity, chronic stress, inflammation, and metabolic imbalance. Stress-sensitive epigenetic regulation has been demonstrated for several genes discussed in this review, including *SLC6A4*, *BDNF*, *FKBP5*, and *TPH2* [9,48,127]. These mechanisms provide a molecular framework for understanding why individuals with similar genetic backgrounds may exhibit markedly different clinical trajectories. Genes that show both genetic effects and environmentally driven epigenetic modifications may therefore represent the most promising targets for translational research.

The polygenic and epigenetically modulated architecture of MDD also offer insight into the high prevalence of psychiatric and somatic comorbidities observed in affected individuals. Depression frequently co-occurs with anxiety disorders, substance use disorders, obesity, metabolic syndrome, cardiovascular disease, and chronic inflammatory conditions. Shared genetic and biological pathways involving stress regulation, neuroplasticity, immune signaling, and metabolic control likely underline these associations [11,114]. The *FTO* gene provides a notable example of this bidirectional relationship, linking metabolic dysregulation to depressive symptoms through epitranscriptomic mechanisms that influence neuronal function [114,117]. These observations support the conceptualization of MDD as a systemic disorder rather than an isolated psychiatric condition. At the same time, such overlap complicates the identification of disorder-specific mechanisms and represents a major challenge for translating genetic findings into clinically actionable tools.

Severe clinical manifestations of depression, including recurrent illness, treatment resistance, and suicidality, may constitute biologically distinct subgroups within the depressive spectrum. Genetic variants in *BDNF*, *NTRK2*, *FKBP5*, *CRHR1*, and *COMT* have been associated with impaired stress adaptation, altered emotional regulation, and increased suicide risk [18,19]. Epigenetic modifications affecting these genes may further amplify vulnerability by disrupting synaptic connectivity, neurotrophic support, and glucocorticoid feedback mechanisms [9,48]. Understanding the genetic and epigenetic architecture of severe depression is therefore critical for identifying high-risk individuals and optimizing therapeutic interventions. These subgroups may represent the most promising candidates for early clinical translation, as genetic effects appear stronger and more biologically consistent than those observed in broadly defined MDD populations.

From a clinical perspective, integrating genetic findings with well-defined molecular mechanisms provides a pathway towards more informative and actionable models of depression. Rather than conceptualizing genetic variants as isolated risk factors, accumulating evidence supports a framework in which combinations of variants across serotonergic, neurotrophic, dopaminergic, and stress-response pathways shape distinct clinical profiles, including symptom dimensions, illness course, and treatment responsiveness. For example, variants affecting synaptic plasticity (e.g., *BDNF*, *NTRK2*), stress sensitivity (e.g., *FKBP5*, *CRHR1*), and monoaminergic signaling (e.g., *SLC6A4*, *COMT*, *SLC6A3*) may jointly define biologically meaningful subtypes of MDD with differential prognostic and therapeutic implications. Emphasizing these convergent mechanisms allows existing genetic data to be repurposed beyond risk prediction, supporting emerging approaches such as biologically informed patient stratification, pathway-based treatment selection, and identification of individuals at elevated risk for recurrence or treatment resistance. This shift from single-variant associations to mechanistic, pathway-oriented interpretation is essential for maintaining the clinical relevance and originality of genetic research in depression and for advancing personalized psychiatry.

Mendelian randomization strategies are increasingly useful for disentangling causal relationships between genetic risk factors, environmental exposures, and disease outcomes. By using genetic variants as instrumental variables, MR helps reduce confounding and reverse causations that often limit observational studies. In cancer and psychiatric research alike, MR can clarify whether associations—such as those between smoking, inflammation, metabolic traits, or immune markers and disease risk—are likely to be causal, thereby strengthening etiological models and informing prevention and therapeutic targeting.

Importantly, while Mendelian randomization is valuable for assessing causal effects of genetic variants in patients, depression is influenced by the cumulative effect of multiple genetic variants, each contributing a small effect size, and polygenic risk scores can capture this complexity. Genetic predisposition interacts with environmental exposures and life course events, such as early-life stress, trauma, or chronic adversity, shaping depression risk and clinical trajectories. Combining MR, polygenic risk assessments and epigenetic, RNA transcript, proteomics and metabolomics investigations will allow a more comprehensive understanding of how genetic and environmental factors jointly influence susceptibility, symptom dimensions, illness course, and treatment response. Causal inference from single variants should always be considered within the broader context of polygenic architecture and environmental modulation, acknowledging the dynamic interplay that drives depression phenotypes. The definition of depression in the context of mood disorders, particularly bipolar disorder, and also in respect to different symptom specifiers, is also an aspect to consider.

Future research should prioritize integrative strategies that combine polygenic risk scores, epigenetic profiling, neuroimaging, and longitudinal clinical data to more fully capture the biological diversity of MDD [11]. Genetic vulnerabilities do not always explain the onset and maintenance of depressed mood and major depressive disorder, as epigenetic, psychosocial, and trauma factors can also contribute to the presence or relapse of symptoms. At the same time, investigations into genetic profiles in depression may also be useful for understanding differences among patients in their potential for resilience and response to different types of treatments. Such approaches may improve the stratification of depressive subtypes and inform personalized treatment decisions, particularly in severe or treatment-resistant cases. However, several practical challenges must be addressed before these findings can be implemented in routine clinical practice, including the standardization of genotyping platforms, replication across diverse populations, establishment of thresholds for clinical actionability, and prospective trials demonstrating added value beyond existing clinical predictors. Given that epigenetic modifications are potentially reversible, they also represent promising therapeutic targets for pharmacological, behavioral, and neuromodulator interventions. Major barriers remain, including small individual effect sizes, limited cross-study harmonization, ethical considerations related to genetic risk disclosure, and the absence of validated clinical algorithms integrating genetic information. Overall, recognizing depression as a disorder emerging from dynamic gene–environment–epigenetic interactions is essential for advancing precision psychiatry and improving long-term clinical outcomes.

## 4. Conclusions

MDD is a complex and diverse condition with a genetic influence. Research shows that variations in genes related to serotonin signaling, neurotrophic factors, dopamine regulation, and stress response contribute to differences among individuals in terms of risk, symptoms, and treatment outcomes. Among these, serotonergic and neurotrophic pathways are supported by the most consistent evidence across studies, whereas dopaminergic and stress-related genetic signals remain more variable and context-dependent. Although no single gene variant can fully explain the risk of depression, combining information from multiple genetic markers, along with environmental, epigenetic, gene expression, protein turn-over and metabolism shaping factors, provides a clearer picture of its causes, risks and different presentations; this multifaceted framework is strongly supported by current evidence.

Recent advances in genomic technology and large collaborative studies are opening new possibilities for personalized psychiatric care. Identifying and confirming clinically meaningful genetic variants can help develop more precise diagnostic tools and targeted treatments. This personalized approach could lead to better treatment responses, fewer side effects, and overall improved quality of life for those with depression. Ongoing linking genetics to brain imaging in more severe pictures, gene expression studies, and behavioral analysis remain vital for turning these findings into diversified practical medical solutions.

## Figures and Tables

**Table 1:** Key genetic variants in serotonergic and kynurenine pathways associated with major depressive disorder, antidepressant response, and neurobiological alterations.

Aspect	Description
Gene	*SLC6A4* (encodes serotonin transporter—SERT)
Polymorphism	5-HTTLPR (insertion/deletion polymorphism in the promoter region of *SLC6A4*)
Alleles	S (short), L (long); triallelic model: L_a, L_g, S
Functional Impact	S allele → reduced transcription, lower SERT expression, less serotonin reuptake
Associated Risk	S/S genotype associated with increased depression risk, especially under stress
Antidepressant Response	S allele carriers show poorer SSRI response and lower remission rates
Neuroimaging Findings	S allele linked to reduced gray matter in amygdala and anterior cingulate; altered fronto-limbic connectivity—brain regions essential for emotion processing and mood regulation
Epigenetic Modifications	Increased methylation of the *SLC6A4* promoter reduces expression and may increase depression risk
Environmental Interaction	Stress, trauma, and inflammation modulate gene expression and serotonin function
Gene	*KMO* (kynurenine monooxygenase) [24]
Polymorphism	Functional SNPs (e.g., rs1053230)
Functional Impact	Shifts tryptophan metabolism toward the kynurenine pathway, reducing availability for serotonin synthesis
Associated Risk	Associated with increased risk of depressive symptoms, particularly in inflammation-related depression
Biological Relevance	Competes with tryptophan hydroxylase–mediated serotonin biosynthesis; influences the balance between neurotoxic and neuroprotective kynurenine metabolites
Gene	*KAT* (kynurenine aminotransferase, e.g., KYAT2) [24]
Polymorphism	Regulatory and coding variants (e.g., rs1480544)
Functional Impact	Modulates conversion of kynurenine to kynurenic acid, affecting neuroprotective mechanisms and serotonin precursor availability
Associated Risk	Linked to depression vulnerability and cognitive symptoms
Biological Relevance	Alters kynurenine pathway flux, indirectly influencing serotonergic neurotransmission

**Table 2:** Key Serotonergic Gene Polymorphisms Associated with Depression and Antidepressant Response.

Gene	Polymorphism (SNP)	Location/Type	Functional Effect/Notes	Associated Clinical Features
*HTR1A* (5-HT1A receptor)	rs6295 (C(-1019)G)	Promoter region	Affects receptor expression; G allele linked to reduced autoregulation of the receptor	Increased risk of depression, anxiety; altered SSRI response
*HTR2A* (5-HT2A receptor)	rs6311 (−1438G/A)	Promoter region	Influences receptor density and binding affinity	Susceptibility to depression, suicidal behavior, antidepressant response variability
*HTR2A* (5-HT2A receptor)	rs6313 (T102C)	Coding region (synonymous)	May affect receptor function viaMessenger ribonucleic acid (mRNA) stability or splicing	Same as above
*HTR1B* (5-HT1B receptor)	rs6296 (G861C)	Coding region (synonymous)	Alters receptor function	Linked to depressive symptoms, impulsivity, suicide risk
*TPH1* (Tryptophan hydroxylase 1)	rs1800532 (A218C)	Intron/regulatory region	Possible effects on enzyme expression; findings mixed	Involved in mood disorders
*TPH2* (Tryptophan hydroxylase 2)	rs4570625 (−703G/T)	Promoter region	Alters enzyme activity and serotonin production	Increased risk of depression, suicidality; influences antidepressant response and stress sensitivity
*TPH2* (Tryptophan hydroxylase 2)	rs7305115	Intronic variant	Linked to altered enzyme function	Same as above

**Table 3:** *BDNF* Polymorphisms Associated with Depression and Treatment Response [50].

SNP ID	Gene	Location/Effect	Notes/Associations
rs7124442	*BDNF*	3′ untranslated region (3′ UTR)	May affect mRNA stability and *BDNF* expression; studied in relation to depression susceptibility and cognitive function.
rs11030104	*BDNF*	Intronic variant	Associated with altered *BDNF* expression and linked to depression and anxiety traits in some populations.
rs2030324	*BDNF*	Promoter region	Implicated in regulation of *BDNF* transcription; some studies suggest links to mood disorders.
rs4923468	*NTRK2*	Gene encoding TrkB receptor	Variants linked to altered receptor function and increased risk of depression and suicidal behavior.
rs2289656	*NTRK2*	Intronic variant	Associated with antidepressant response variability and neuroplasticity.
rs11030107	*BDNF*	Intronic variant	Explored in relation to neuroticism and depressive symptoms.
rs6265	*BDNF*	Coding region (missense variant)	Increased risk of depression; Met allele carriers show lower plasma BDNF levels and a less favorable clinical course, including poorer treatment response and greater chronicity.

## Data Availability

No new data were created or analyzed in this study. Data sharing is not applicable to this article. The authors have reviewed and edited the output and take full responsibility for the content of this publication.
